# Supplementary material for: Lung cancer organoids analyzed on microwell arrays predict drug responses of patients within a week
Source: Nat Commun. 2021 May 10;12:2581. doi: 10.1038/s41467-021-22676-1 (PMC8110811; doi:10.1038/s41467-021-22676-1)
Supplement: Supplementary file 1 — Supplementary Information [file 41467_2021_22676_MOESM1_ESM.pdf]

Supplementary information for

Lung cancer organoids analyzed on microwell arrays predict drug responses of patients within a week

Yawei Hu[1,#], Xizhao Sui[2,#], Fan Song[3], Yaqian Li[3], Kaiyi Li[1], Zhongyao Chen[1], Fan Yang[2], Xiuyuan Chen[2], Yaohua Zhang[3], Xianning Wang[4], Qiang Liu[5], Cong Li[6], Binbin Zou[6], Xiaofang Chen[3,7,*], Jun Wang[2,*] and Peng Liu[1,*]

[1] Department of Biomedical Engineering, School of Medicine, Tsinghua University, Beijing, 100084, China

[2] Department of Thoracic Surgery, People's Hospital, Peking University, Beijing, 10044, China

[3] Key Laboratory for Biomechanics and Mechanobiology of Ministry of Education, School of Biological Science and Medical Engineering, Beihang University, Beijing, 100083, China

[4] Beijing OrganoBio Corporation, Beijing, 102206, China

[5] Department of Thoracic Surgery, Beijing Haidian Hospital, Beijing, 100080, China

[6] Beijing NeoAntigen Biotechnology Co. Ltd, Beijing, 102206, China.

[7] Interdisplinary Institute of Cancer Diagnosis and Treatment, Beijing Advanced Innovation Centre for Biomedical Engineering, Beihang University, Beijing, 100083, China.

[#] Joint first authors with equal contributions

[*] Joint corresponding authors:

Peng Liu, Department of Biomedical Engineering, School of Medicine, Tsinghua University, Haidian District, Beijing, 100084, China. Phone: +86-10-62798732, fax: +86-10-62798732, email: pliu@tsinghua.edu.cn

Xiaofang Chen, School of Biological Science and Medical Engineering, Beihang University, Haidian District, Beijing, 100191, China. Phone: +86-10-82315554, fax: +86-10-82315554, email: xfchen@buaa.edu.cn

Jun Wang, Department of Thoracic Surgery, People's Hospital, Peking University, Xicheng District, Beijing, 100044, China. Phone:+86-10-88325952, email: wangjun@pkuph.edu.cn

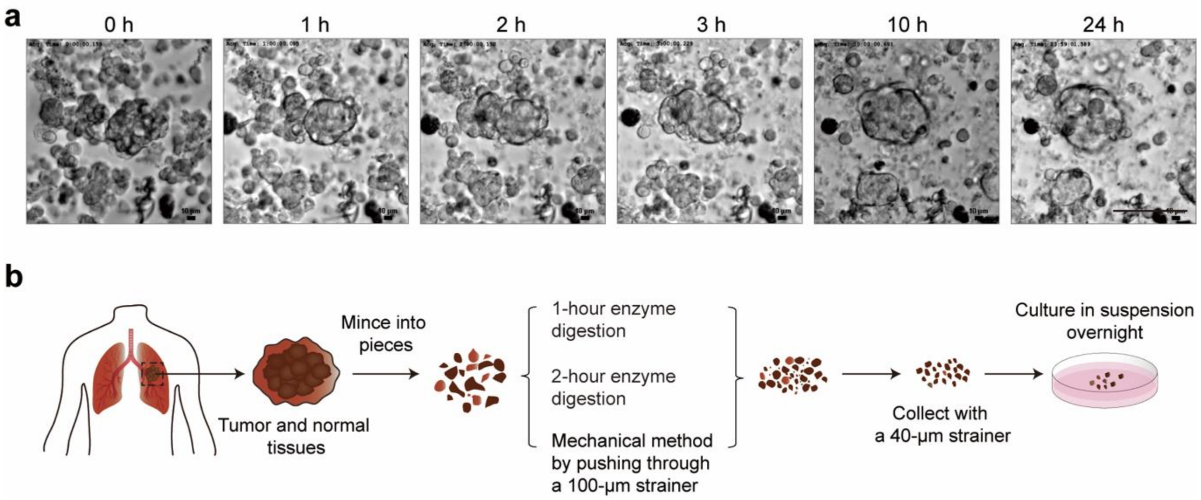

Supplementary Fig. 1 Generation of the lung cancer organoids (LCOs).

a Bright-field images tracking the formation of lung cancer organoids (LCOs) from cell clusters during the overnight suspension culture. 0h identifies the time the cell clusters isolated from the tumor tissue and suspended in lung cancer organoid culture media (LCOM). Scale bar, 200 μm. The experiments are repeated for 3 times. **b** Procedure diagram illustrating the comparison among different sample processing strategies, including mechanical, 1-h, and 2-h enzymatic digestions.

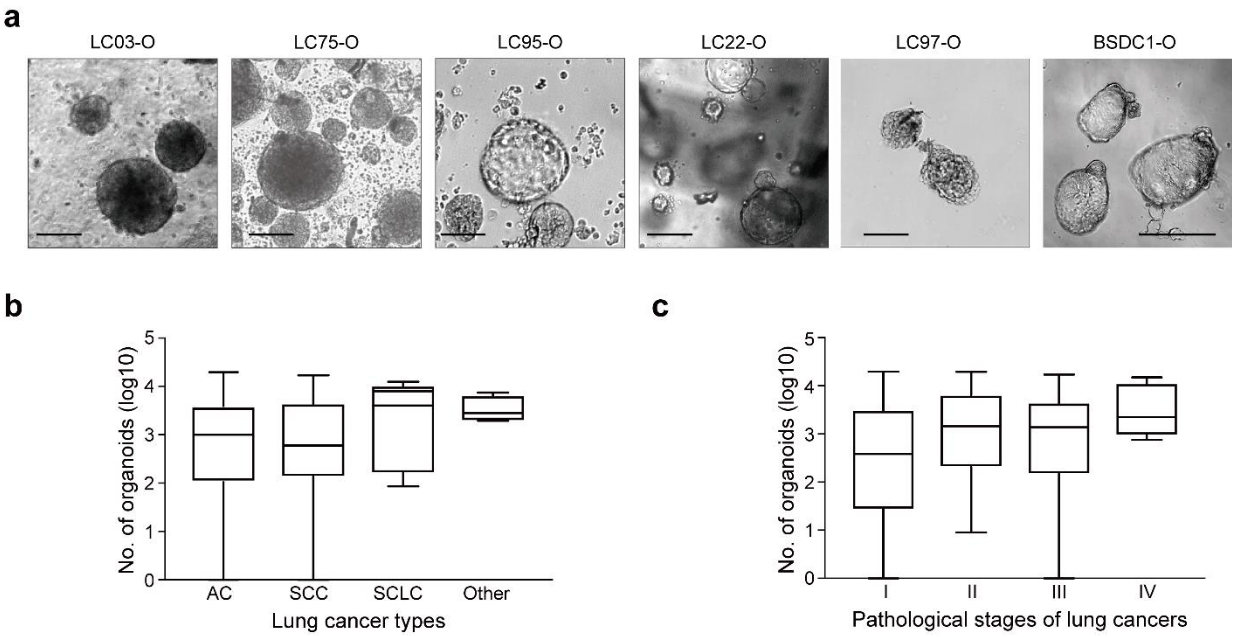

Supplementary Fig. 2 Characterization of the mechanical sample processing method.

a Bright-field images showing three types of morphologies of LCOs: solid spheres (LC103-O and LC75-O), luminal spheres (LC95-O and LC22-O), and loosely connected granular sheets (LC97-O). The image (BSDC1-O) on the right shows the luminal organoids generated from a bronchial salivary gland carcinoma. Scale bars: 200 µm. The experiments are repeated in 142 patient samples. **b and c.** Bar graphs showing the numbers of organoids generated from tumor samples of different lung cancer types (b) and disease stages (c). Note the wide range of the LCO numbers generated from individual tumor samples. AC: adenocarcinoma, SCC: squamous cell carcinoma, SCLC: small cell lung cancer, Other: other lung cancer types. Detailed information about these samples can be found in Supplementary Table 1. (n=69, 23, 5, 4 biologically independent samples for AC, SCC, SCLC and other in **b**; n=40, 21, 32, 8 biologically independent samples for stages I, II, III and IV in **c.** The centre line represents the median value. The bounds of box represent the median values of the upper half and the lower half. The bounds of whiskers represent the maxima and the minima)

a

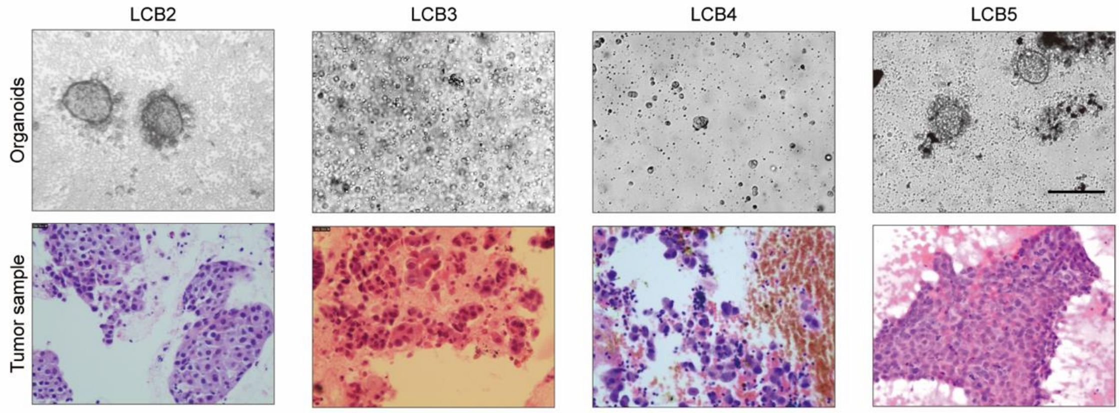

b

Patient No.	Pathological type	Sampling method	Localization	Pathological stage	TNM	Bulky/discrete lymph node metastasaes	Treatment	Lymph node diameter > 2cm	No. of LCOs
LCB1	AC	EBUS-TBNA	lung	ⅣB	T2aN2M1c	discrete	None	Yes	641
LCB2	AC		lymph node	ⅢB	T3N3M0	discrete	None	No	845
LCB3	AC		lymph node	ⅢA	T2aN2M0	discrete	None	No	0
LCB4	AC		lymph node	ⅢB	T3N2M0	Bulky	None	Yes	0
LCB5	SCC		lymph node	ⅢA	T1bN2M0	discrete	None	No	32
LCB6	AC		lymph node	ⅣB	T2bN3M1c	discrete	None	No	247
LCB7	AC		lymph node	ⅣB	T2bN3M1c	Bulky	None	Yes	0
LCB8	SCC		lymph node	ⅣA	T2aN2M1b	discrete	None	No	0

Supplementary Fig. 3 Generation of LCOs from endobronchial ultrasound-guided transbronchial needle aspiration (EBUS-TBNA) samples.

a Bright-field images of cell cluster suspensions 24 hours post processing and H&E staining images of the corresponding EBUS-TBNA samples. LCOs with the solid sphere morphology were generated from samples with large pieces of unbroken tumor tissues (LCB2 and LCB5), while seriously damaged samples dispersed into single cells (LCB3 and LCB4). Scale bar, 200 μm. The experiments are repeated in 8 patient samples. **b** Pathological information of eight EBUD-TBNA samples.

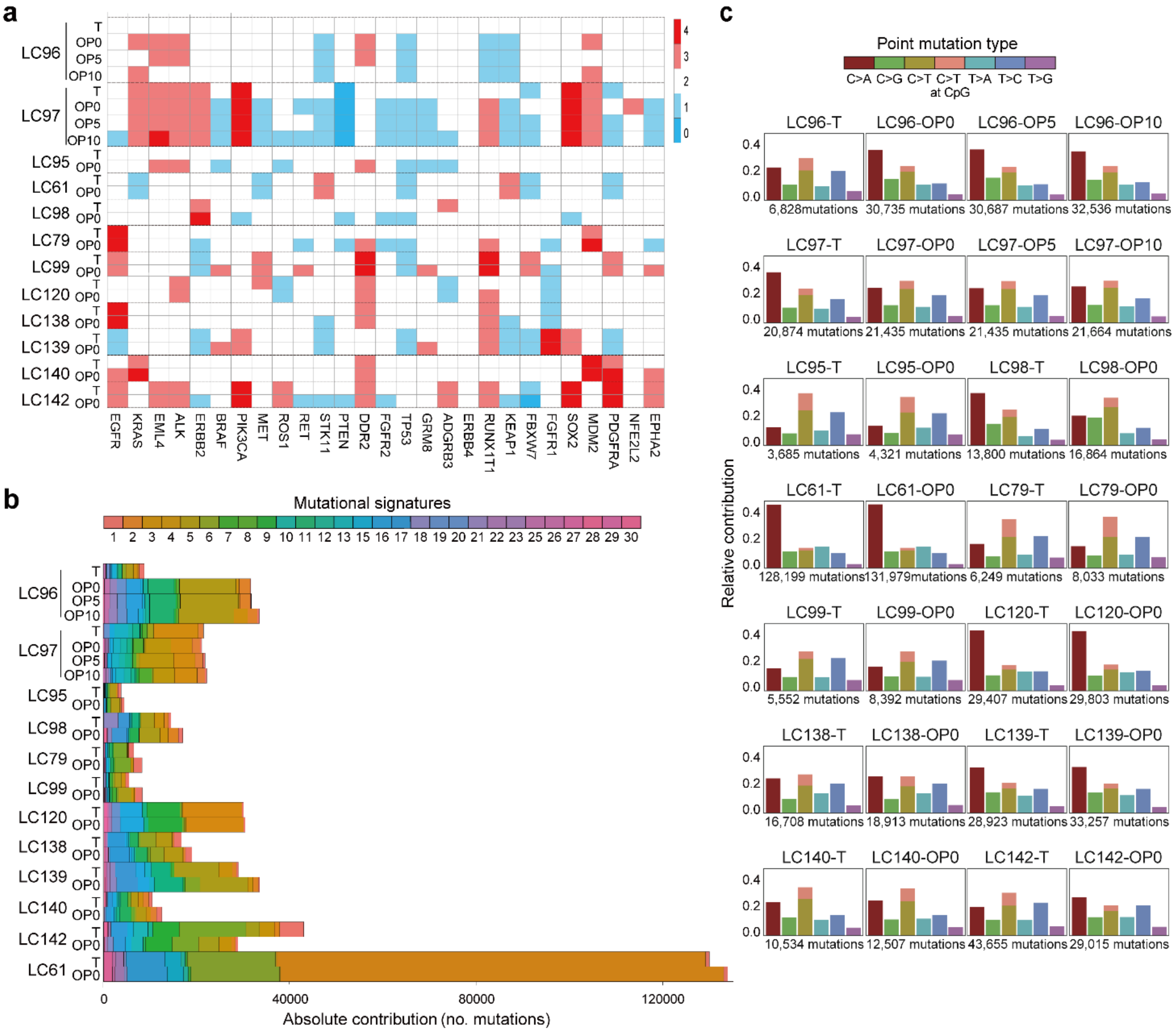

Supplementary Fig. 4 Analysis of genetic alternations in lung cancer organoids.

a Heat map showing gene specific CNVs of the tissue-organoid pairs. **b** Stacked bar graphs showing the total mutation loads per mutational signature in all the patient tissue-organoid pairs. Note the heterogeneity among different patient samples and the consistency between the original tumor tissues and the derived organoids at passage 0. **c**. Bar graph showing the relative contributions of the point mutation types for all the patient tissue-organoid pairs. Mutation types are conserved within tissue-organoid pairs.

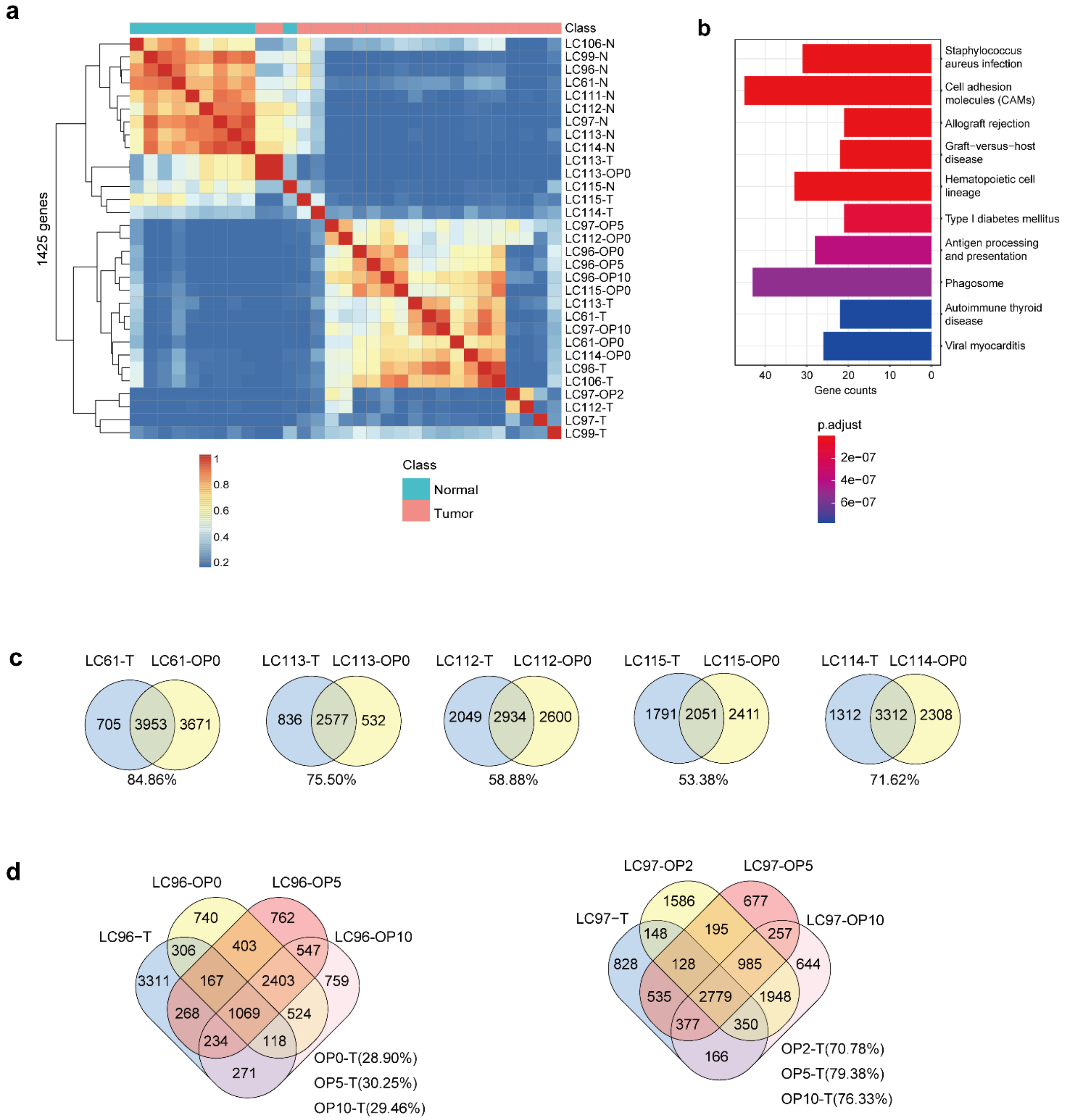

Supplementary Fig. 5 Gene expression analysis of the LCOs by RNA-seq.

a Correlation heat map the LCOs, the original tumor tissues and the paracancer tissues. Spearman corrections were calculated for all sample-pairs. Cells are color-coded by the Spearman correlation value. (T denotes the tumor tissue, O denotes the organoid, and N denotes the normal tissue). **b** Barplot of the most enriched KEGG pathways between the LCOs and the parental tumor tissues. **c** Van diagram showing the overlap in gene expression between the LCOs and the original tumor tissues (Genes that have the similar level of expression with the corresponding normal tissue were not included). **d** Van diagram illustrating the overlap in gene expression between the passaged

organoids and the tumor tissues of LC96 and LC97 (Genes that have the similar level of expression with the corresponding normal tissue were not included). OP0: passage 0 organoids. OP2: passage 2 organoids, OP5: passage 5 organoids, and OP10: passage 10 organoids.

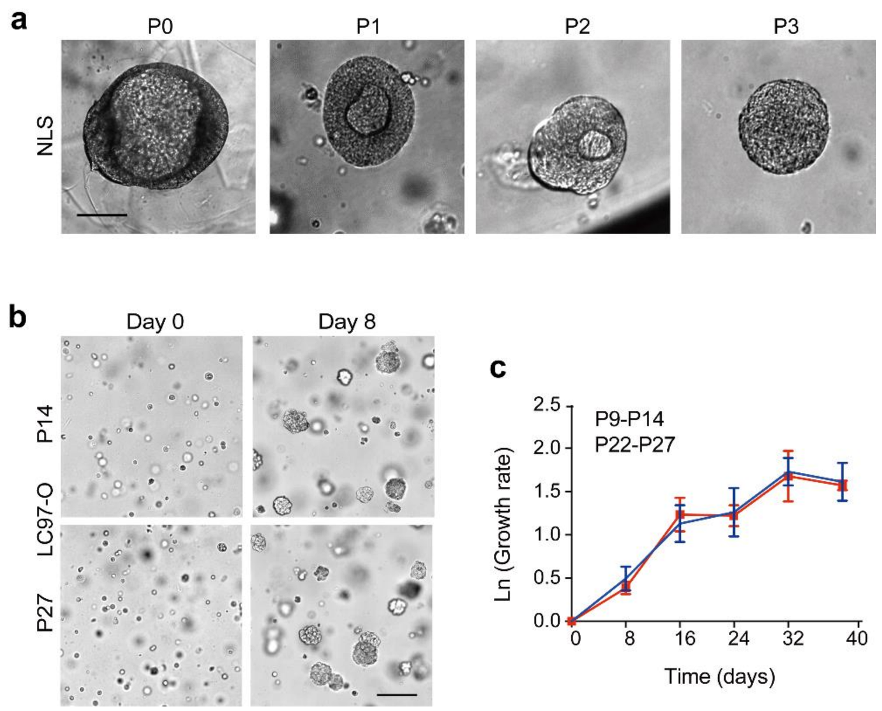

Supplementary Fig. 6 Passage and growth of the LCO lines.

a Bright-field images showing the morphologies of spheroids derived from normal lung tissue (NLS) at different passages. Scale bar, 100 µm. The experiments are repeated in 3 patient samples. **b** Bright-filed images of the organoid line LC97-O at day 0 and day 8 after passaging. P14: passage 14, P27: passage 27. Scale bar, 200 µm. The experiments are repeated for 3 times. **c** The line chart showing the growth rate of LC97-O at different passages. There was no difference in the growth rates between the organoids at the high passage (P22-P27) and the low passage (P9-P14). The organoids were passaged every 8 days. (n=3 biologically independent cells. Data are presented as mean ± SD).

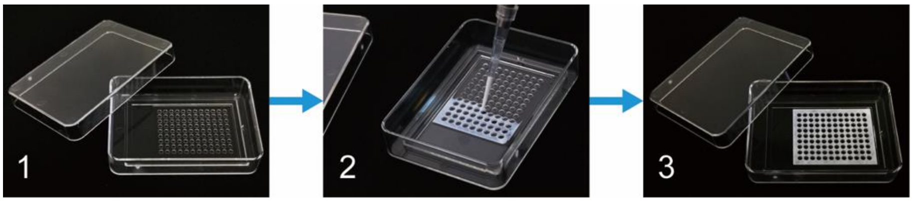

Supplementary Fig. 7 Fabrication procedure of the Integrated superhydrophobic microwell array chip (InSMAR-chip). The first step is to clean the integrated microwell array chip manufactured by injection molding, the second is to add the home-made superhydrophobic paint onto the surface of the array, and the third is to air-dry and autoclave the integrated superhydrophobic microwell array chip.

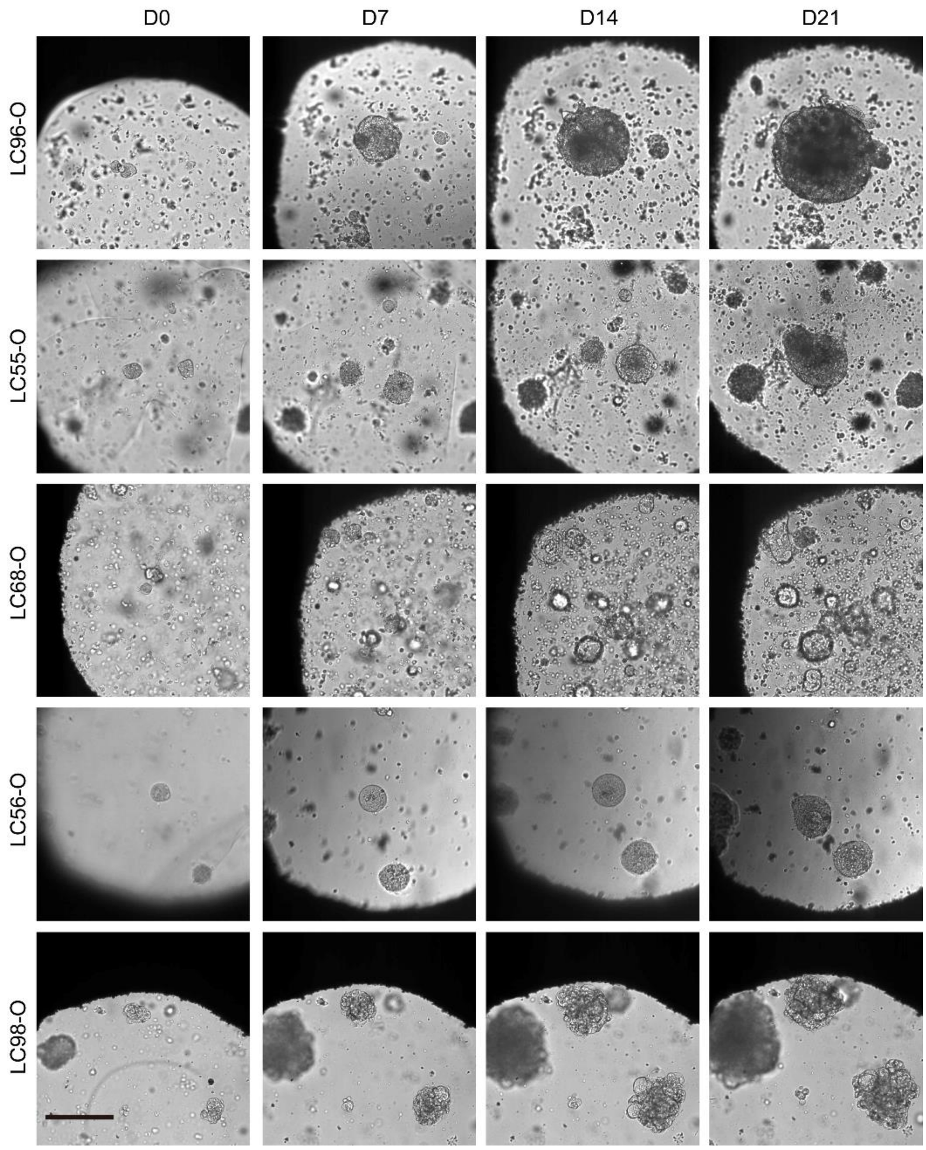

Supplementary Fig. 8 Tracing of LCOs cultured on the InSMAR-chip. Continuous growth can be seen from day 0 to day 21. Scale bar: 200 μm. The experiments are repeated for 3 times.

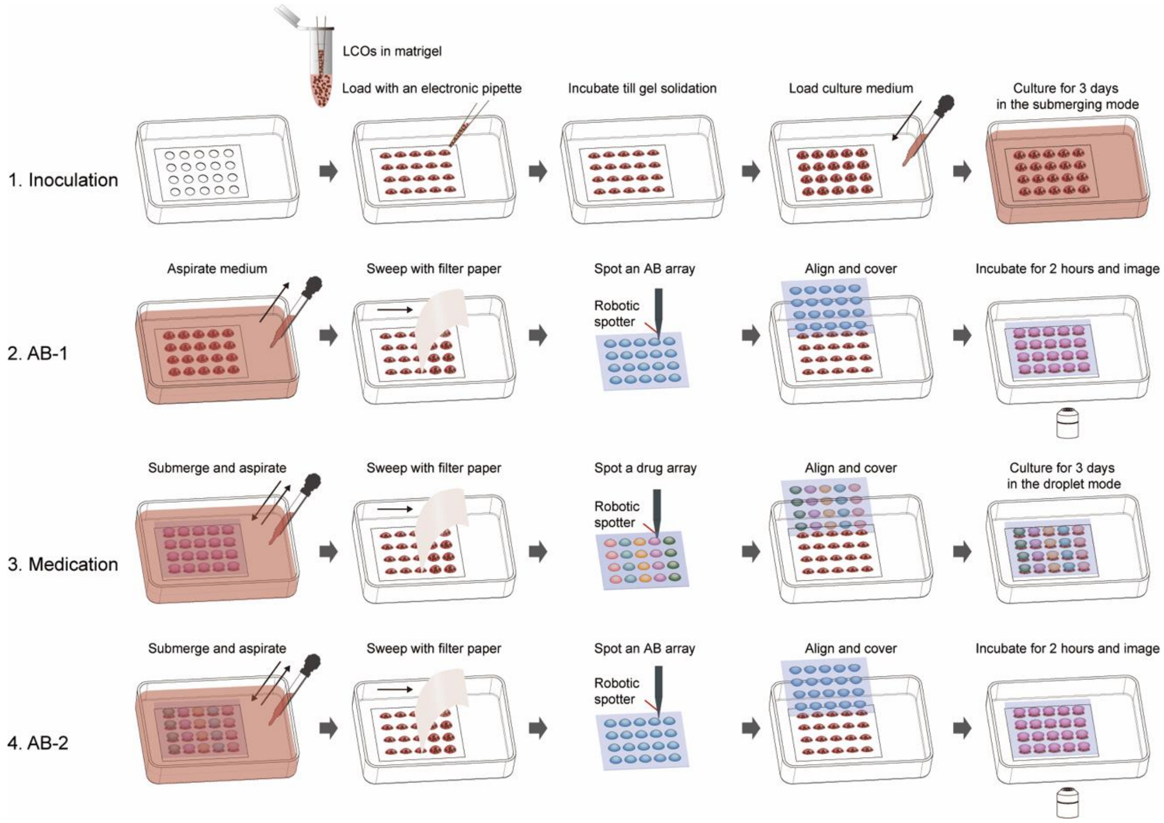

Supplementary Fig. 9 Procedure of the one-week drug sensitivity tests performed on the SMAR-chip.

The procedure includes four major steps: the inoculation, the first cell viability test using the alamarBlue^{TM}, the medication, and the second cell viability test using the alamarBlue^{TM}. The detailed description can be found in the section of Methods.

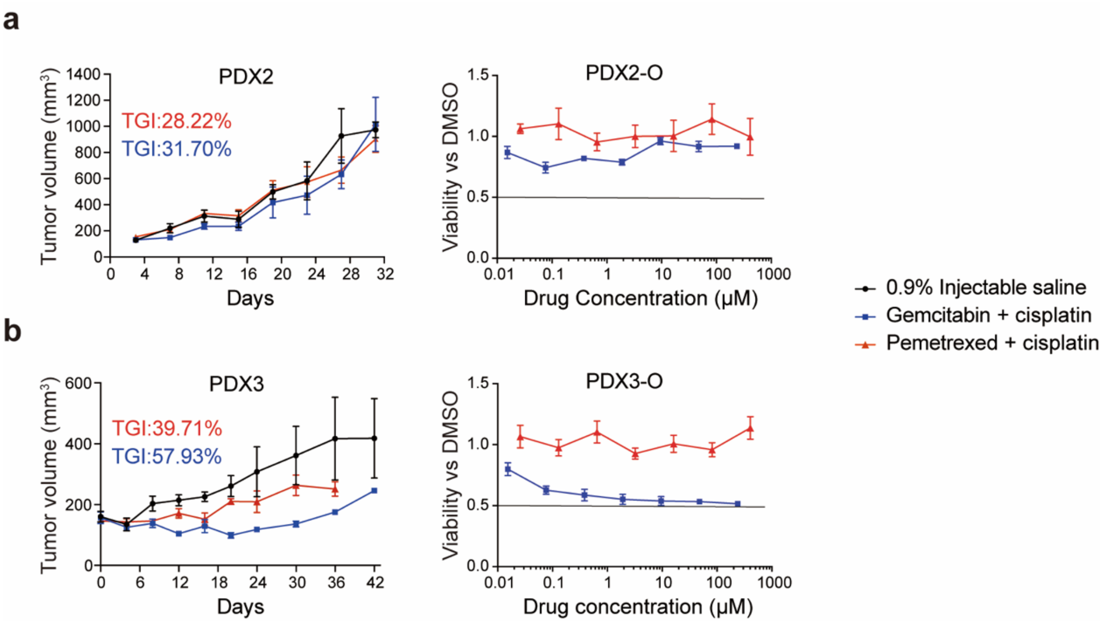

Supplementary Fig. 10 Drug response comparison between the PDX mice and the PDX-derived organoids. The Line charts on the left showing the growth of xenograft tumors in PDX mice exposed to chemotherapies. TGI is the tumor growth inhibition (n=3 biologically independent animals. Data are presented as mean ± SD). The line charts on the right showing the viabilities of the PDX-derived organoids exposed to increasing concentrations of the same drugs (n=3 biologically independent cells. Data are presented as mean ± SD).

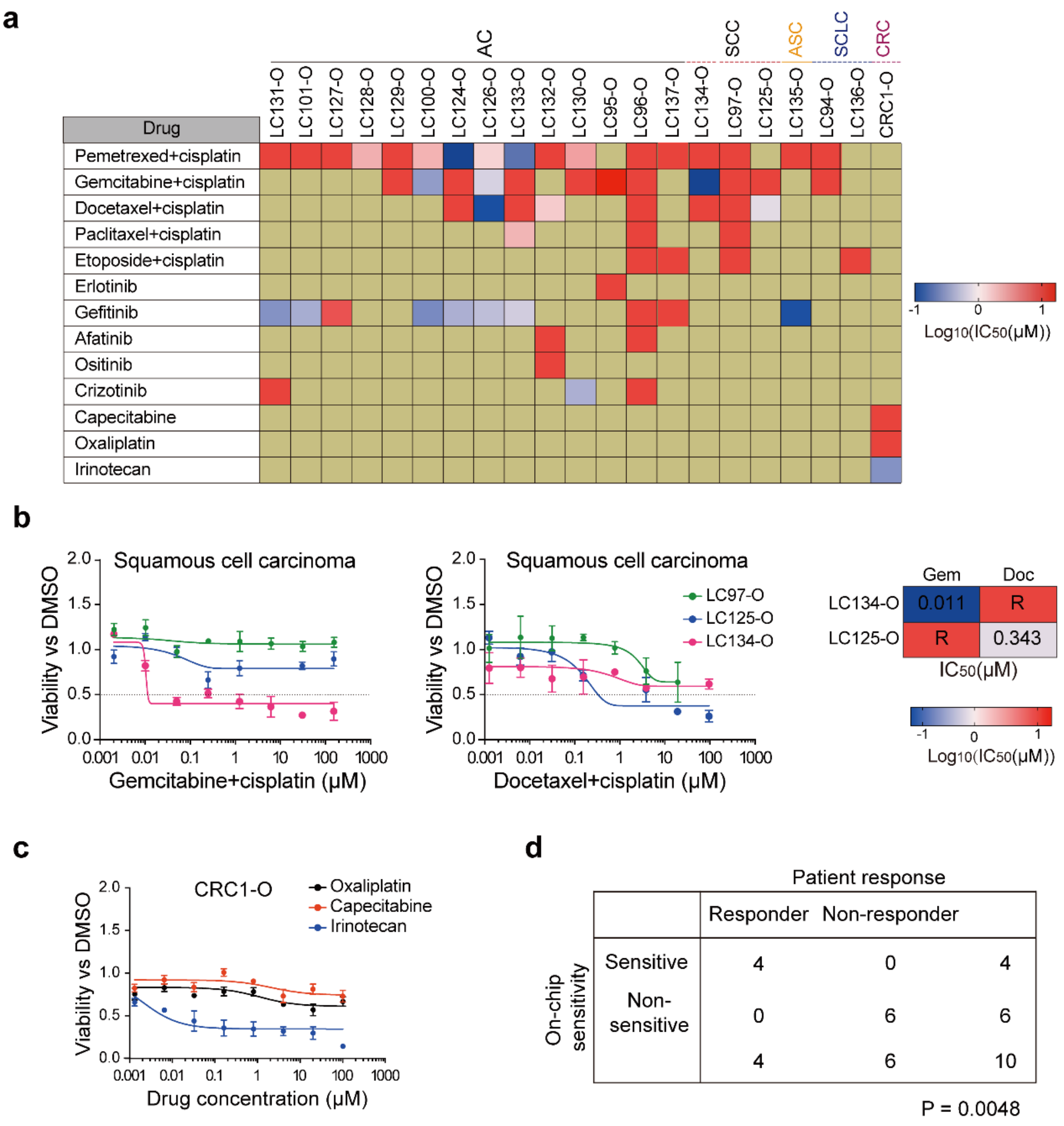

Supplementary Fig. 11 Summary of the responses of 21 organoid lines to anti-cancer drugs.

a Heat map illustrating the sensitivities of 21 tumor organoid lines to chemotherapies and targeted therapies. The sensitivity is denoted by the $\log_{10}(IC_{50}(\mu M))$ value. **b** Responses of organoids derived from lung squamous cell carcinomas to the gemcitabine+cisplatin (left panel) and docetaxel+cisplatin (middle panel). LC134-O and LC135-O have completely different responses to these therapies (right panel). (n=3 biologically independent cells. Data are presented as mean ± SD). **c** Fitted dose response curves of the colorectal cancer organoid (CRC1-O) to three chemotherapy drugs (n=3 biologically independent cells. Data are presented as mean ± SD). **d** Correlation responses between the patients and the on-chip drug sensitivity results.

Supplementary Table 1: Mutations in the EGFR and ALK gene in 12 tumor samples (obtained from the clinical data).

Sample ID	EGFR mutation	ALK mutation	Targeted therapy drugs tested
LC95	None	None	Erl
LC96	None	None	Gef, Afa, Cri
LC100	Exon 19 indel	None	Gef
LC101	Exon 21,P.L858R	None	Gef
LC124	Exon 18,P.G719A	None	Gef
LC126	Exon 21,P.L858R	None	Gef
LC137	None	None	Gef
LC127	None	None	Gef
LC130	None	EML4-ALK(E6-A20)	Cri
LC131	Exon 19 indel	None	Gef, Cri
LC132	Exon 21,P.L858R	None	Afa, Oxi
LC133	Exon 19 indel	None	Gef
Erl: erlotinib, Gef: gefitinib, Afa: afatinib, Oxi: Oxitinib, Cri: Crizotinib.			

Supplementary Table 2. Recipe of the lung cancer organoid culture media (LCOM)

Regents	Source	Catalog No.	Final concentration
DMEM/F12	ThermoFisher Scientific	11320-033	
GlutaMAX	Gibco	35050-061	1%(v/v)
HEPES	Gibco	15630-080	10 mM
Penicillin/Streptomycin	Hyclone	SV30010	1%(v/v)
B-27 Supplement	Invitrogen	17504044	2% (v/v)
N2 Supplement	Invitrogen	17502048	1% (v/v)
Nicotinamide	Selleckchem	1899	5 mM
N-Acetyl-L-cysteine	Selleckchem	S1632	1 mM
Y-27632	Selleckchem	S1049	10 μM
EGF	Peprotech	AF-100-15	50 ng/mL
SB202190	Selleckchem	S1077	3 μM
A83-01	Tocris	2939	5 μM
Forskolin	Selleckchem	2449	10 μM
Dexamethasone	Selleckchem	1322	3 nM

Supplementary Table 3. Antibody information

Target	Source	Catalog No.	Dilution
Rabbit monoclonal anti-Akt(pan)	CST	4691	1: 300
Rabbit monoclonal anti-phospho-Akt(Ser473)	CST	4060	1: 100
Rabbit monoclonal anti-p44/42 MAPK (Erk1/2)	CST	4695	1: 250
Rabbit monoclonal anti-phospho-p44/42 MAPK (Erk1/2)	CST	4370	1: 400
Anti-TTF-1	ORIGENE	ZM-0270	1: 200
Anti-CK-7	ORIGENE	ZM-0472	1: 200
Anti-P40	ORIGENE	ZM-0406	working fluid
Anti-cytokeratin 5/6	ORIGENE	ZM-0313	1:200
Anti-P63	ORIGENE	ZM-0071	working fluid
IgG polymer	ORIGENE	PV6000D2	working fluid

Supplementary Table 4. Primer sequence for real-time PCR detection

Gene		Primer sequence	Amplicon length
Bcl-2	Forward	5’ -GGATCCAGGATAACGGAGGCTGG-3’	169 bp
	Reverse	5’ -CCACC AGGGCCAAAC TGAGC-3’	
Beclin-1	Forward	5′-ATCCTGGACCGTGTCACCATCCAGG-3′	363 bp
	Reverse	5′-GTTGAGCTGAGTGTCCAGCTGG-3′	
LC-3	Forward	5′-GAAGATGTCCGACTTATTCGAGAG-3′	352 bp
	Reverse	5′-ACTCTCATACACCTCTGAGATTGG-3′	
β-actin	Forward	5’-TTCAGGTTTACTCACGTCATCC-3’	317 bp
	Reverse	5’-CAAATGCGGCATCTTCAAACCC-3’	

Supplementary Table 5. Chemotherapy and targeted therapy drugs

Chemicals	Source	Catalog No.
Pemetrexed	Macklin Biochemicals, China	150399-23-8
Gemcitabine	Energy Chemical, China	95058-81-4
Docetaxel	HarveyBio	114977-28-5
Paclitaxel	TargetMol	33069-62-4
Etoposide	Macklin Biochemicals, China	33419-42-0
Cisplatin	Energy Chemical, China	15663-27-1
Carboplatin	HarveyBio	41575-94-4
Gefitinib	Pharmacodia, China	184475-35-2
Afatinib	TargetMol	850140-73-7
Erlotinib	TargetMol	183321-74-6
Osimertinib	TargetMol	1421373-66-1
Capecitabine	TargetMol	154361-50-9
Oxaliplatin	TargetMol	61825-94-3
Irinotecan	TargetMol	97682-44-5
Crizotinib	TargetMol	877399-52-5

Supplementary Table 6. Reagents and critical kits

Reagent or kit	Source	Catalog No.
Matrigel	Corning BD	354230
Cultrex Organoid Harvesting Solution	R&D Systems	3700-100-01
RNA Later	QIAGEN	76104
4 % PFA	LEAGENE	DF0135
alamarBlue Cell Viability Reagent	Invitrogen	DAL1025
Phosphate Buffered Saline (1×)	HyClone	SH30256.01
DEPC-Treated Water	Ambion	1704009
Cryopreservation Medium (Serum Free)	CELLBANKER2	11891
10×TBST	Solarbio	T1081
10×Transfer Buffer	Solarbio	D1060-500
10×Tris-Glycine-SDS Buffer	LABLEAD	T7777
Bovine Serum Albumin	Sigma-Aldrich	V900933-100G
0.25% Trypsin-EDTA (1×）	Gibco	25200-072
DAB Substrate buffer	ORIGENE	PV-6000D3
DAB chromogenic solution	ORIGENE	PV-6000D4
1xHBSS	LEAGENE	CC0030
protease inhibitor cocktail	Sigma-Aldrich	P9599-1ML
Phosphatase Inhibitor Cocktail 2	Sigma-Aldrich	P5726-1ML
RIPA Buffer	Sigma-Aldrich	R0278-50ML
Fibrinogen	Sigma-Aldrich	Cat#9001-32-5
Thrombin	Solarbio	Cat#9002-04-4
AMPure XP	Beckman Coulter	Cat#A63881
RNase	Thermo Fisher Scientific	EN0601
POWRUP SYBR MASTER MIX	Thermo Fisher Scientific	A25742
BeyoClick™ EdU-549 Cell Proliferation Kit	Beyotime	C0078S
Calcein-AM/PI Double Stain Kit	YEASEN	40747ES76
BCA Protein Assay Kit	CWBIO	CW0014S
Mammalian Protein Extraction Kit	CWBIO	CW0889S
SDS-PAGE Gel Kit	CWBIO	CW0022S
eECL Western Blot Kit	CWBIO	CW0049
DNeasy Blood&Tissue Kit	QIAGEN	69504
RNeasy Mini Kit	QIAGEN	74104
Dynabeads mRNA DIRECT Kit	Invitrogen	61011
Poly(A)mRNA Magnetic Isolation Module	NEBNext	E7490L
Ultra Ⅱ RNA Library Prep Kit	NEBNext	E7770S
Ultra Ⅱ DNA Library Prep Kit	NEBNext	E7645S
Qubit RNA HS Assay Kit	Invitrogen	Q32852
Qubit dsDNA HS Assay Kit	Invitrogen	Q32854
Multiplex Oligos for illumina	NEBNext	E7335L
ProtoScript II First Strand cDNA Synthesis Kit	NEGNext	E6560L
