# Supplementary material for: Lung cancer organoids analyzed on microwell arrays predict drug responses of patients within a week
Source: Nat Commun. 2021 May 10;12:2581. doi: 10.1038/s41467-021-22676-1 (PMC8110811; doi:10.1038/s41467-021-22676-1)
Supplement: Supplementary file 3 — Description of Additional Supplementary Files [file 41467_2021_22676_MOESM3_ESM.docx]

File Name: Supplementary Information
Description: Supplementary Fig. 1 to 11 and Supplementary table 1 to 6

File Name: Supplementary Data 1

Description: Pathological information of all the resected samples

File Name: Supplementary Data 2

Description: Clinical characteristics of patient samples used in the on-chip drug sensitivity tests

File Name: Source Data

Description: Quantitative data of the figures

File Name: Supplementary Video 1

Description: Formation of multicellular organoids from suspension of cell clusters

File Name: Supplementary Video 2

Description: Formation of droplet array on the InSMAR-chip
