# Supplementary material for: Lung cancer organoids analyzed on microwell arrays predict drug responses of patients within a week
Source: Nat Commun. 2021 May 10;12:2581. doi: 10.1038/s41467-021-22676-1 (PMC8110811; doi:10.1038/s41467-021-22676-1)
Supplement: Supplementary file 8 — Reporting Summary [file 41467_2021_22676_MOESM8_ESM.pdf]

nature research

Corresponding author(s): *Double-blind peer review submissions: write DBPR and your manuscript number here instead of author names.*

Last updated by author(s): *YYYY-MM-DD*

Reporting Summary

Nature Research wishes to improve the reproducibility of the work that we publish. This form provides structure for consistency and transparency in reporting. For further information on Nature Research policies, see our Editorial Policies and the Editorial Policy Checklist.

Statistics

For all statistical analyses, confirm that the following items are present in the figure legend, table legend, main text, or Methods section.

n/a	Confirmed	
☐	☒	The exact sample size (*n*) for each experimental group/condition, given as a discrete number and unit of measurement
☐	☒	A statement on whether measurements were taken from distinct samples or whether the same sample was measured repeatedly
☐	☒	The statistical test(s) used AND whether they are one- or two-sided<br>*Only common tests should be described solely by name; describe more complex techniques in the Methods section.*
☒	☐	A description of all covariates tested
☒	☐	A description of any assumptions or corrections, such as tests of normality and adjustment for multiple comparisons
☐	☒	A full description of the statistical parameters including central tendency (e.g. means) or other basic estimates (e.g. regression coefficient) AND variation (e.g. standard deviation) or associated estimates of uncertainty (e.g. confidence intervals)
☐	☒	For null hypothesis testing, the test statistic (e.g. *F*, *t*, *r*) with confidence intervals, effect sizes, degrees of freedom and *P* value noted<br>*Give P values as exact values whenever suitable.*
☒	☐	For Bayesian analysis, information on the choice of priors and Markov chain Monte Carlo settings
☒	☐	For hierarchical and complex designs, identification of the appropriate level for tests and full reporting of outcomes
☒	☐	Estimates of effect sizes (e.g. Cohen's *d*, Pearson's *r*), indicating how they were calculated

Our web collection on statistics for biologists contains articles on many of the points above.

Software and code

Policy information about availability of computer code

Data collection	No software was used
Data analysis	Genome Analysis Toolkit (GATK) v 4.1.0.0 for DNA sequence analysis, Burrows-Wheeler Alignment with maximal exact matches (BWA-MEM) v0.7.16a for DNA sequence alignment, edgeR package(v3.24.3) for transcript level analysis, ImageJ bundled with Java 1.8.0_172 for image analysis, SAMBLASTER v.0.1.24, SAM tools v1.9, Sclust v1.0, CNVkit v0.9.3, R software (v3.0), FastQC (v0.11.5), Cutadapt v2.1, STAR software (2.5.3a), feature Counts (Version 1.5.0-p1), Graph Pad Prism8 (GraphPad Software). Adobe Illustrator CC 2019 software.

For manuscripts utilizing custom algorithms or software that are central to the research but not yet described in published literature, software must be made available to editors and reviewers. We strongly encourage code deposition in a community repository (e.g. GitHub). See the Nature Research guidelines for submitting code & software for further information.

Data

Policy information about availability of data

All manuscripts must include a data availability statement. This statement should provide the following information, where applicable:

- Accession codes, unique identifiers, or web links for publicly available datasets
- A list of figures that have associated raw data
- A description of any restrictions on data availability

The raw sequence data reported in this paper have been deposited in the Genome Sequence Archive (Genomics, Proteomics & Bioinformatics 2017) in National Genomics Data Center (Nucleic Acids Res 2020), Beijing Institute of Genomics (BIG), Chinese Academy of Sciences, under accession number HDAC000233 at https://bigd.big.ac.cn/gsa-human/browse. The sequence data is also available from the corresponding author (pliu@tsinghua.edu.cn). The source data underlying Fig. 1b, e, g, h, Fig. 3g, h, Fig. 4g, j, Fig. 5a, b, d, Fig. 6a, c, f and Supplementary Fig. 2b, c, Supplementary Fig. 6c, Supplementary Fig. 10 and Supplementary Fig. 11 are provided as a Source data file. All the other data supporting the findings of this study are available within the article and its supplementary information files and from the

corresponding author upon reasonable request. A reporting summary for this article is available as a Supplementary Information file.

Field-specific reporting

Please select the one below that is the best fit for your research. If you are not sure, read the appropriate sections before making your selection.

- [x] Life sciences
- [ ] Behavioural & social sciences
- [ ] Ecological, evolutionary & environmental sciences

For a reference copy of the document with all sections, see nature.com/documents/nr-reporting-summary-flat.pdf

Life sciences study design

All studies must disclose on these points even when the disclosure is negative.

Sample size	We processed all the lung cancer samples (142) we received from the hospital during the peroid of the study (from Sep. 2017 to Dec. 2019) to generate lung cancer organoids. The sample size is large enough to demonstrate the reliability of the sample processing method (n=142 biologically independent samples). We used 21 samples to validate the on chip drug sensitivity test which covered the main lung cancer types, including lung adenocarcinoma (EGFR mutated and wild type), squamous cell lung cancer and small cell lung cancer. In 10 of the 21 samples, the clinical data can be campared with the on chip drug sensitivity test results, achieving 100% accuracy and specificity (Fisher's exact test, P=0.0048).
Data exclusions	No data were excluded from the analysis
Replication	All the experiments were repeated for more than three times independently. We performed the LCO generation experiments for 142 times and achieved a success rate of 79%. We passaged 20 LCO lines and 15 of them were successfully passaged for at least 3 times. We performed the chip fabrication for more than 100 times and 100% of the experiments were successful. We performed the on chip drug sensitivity tests for more than 20 times and all the experiments were successful. We include detailed protocol and source of reagents for the generation of lung cancer organoids., the chip fabrication and the on chip drug sensitivity tests. These experiments can be reproduced easily.
Randomization	The major goal of the study is to develop a technology for lung cancer organoid-based drug sensitivity test. So the experimental samples were not grouped and randomlized.
Blinding	Blinding is not relevant to this study since the samples were not devided into groups.

Reporting for specific materials, systems and methods

We require information from authors about some types of materials, experimental systems and methods used in many studies. Here, indicate whether each material, system or method listed is relevant to your study. If you are not sure if a list item applies to your research, read the appropriate section before selecting a response.

Materials & experimental systems

n/a	Involved in the study	
[ ]	[x]	Antibodies
[x]	[ ]	Eukaryotic cell lines
[x]	[ ]	Palaeontology and archaeology
[ ]	[x]	Animals and other organisms
[ ]	[x]	Human research participants
[x]	[ ]	Clinical data
[x]	[ ]	Dual use research of concern

Methods

n/a	Involved in the study	
[x]	[ ]	ChIP-seq
[ ]	[x]	Flow cytometry
[x]	[ ]	MRI-based neuroimaging

Antibodies

Antibodies used	Primary antibodies: histology and imaging_anti-TTF-1 (#ZM-0270, ORIGENE), anti-cytokeratin 7 (CK7; #ZM-0472, ORIGENE), anti-p63 (#ZM-0071, ORIGENE), anti-cytokeratin 5/6 (CK5/6; #ZM-0313, ORIGENE), anti-p40 (#ZM-0406, ORIGENE), phosphorylated Akt (pAkt; #4060, Cell Signaling Technology), Akt (#4691, Cell Signaling Technology), phosphorylated Erk1/2 (pErk;#4370, Cell Signaling Technology), Erk 1/2 (Erk;#4695, Cell Signaling Technology). Secondary antibodies: Histology and imaging_Enzyme-labeled goat Anti-Rouse / Rabbit IgG Polymer (#PV-6000D2, ORIGENE).
Validation	Primary antibodies: histology and imaging_anti-TTF-1(1:200 dilutions, anti-mouse), anti-cytokeratin 7 (CK7; 1:400 dilutions, anti-mouse), anti-p63(1:200 dilutions, anti-mouse), anti-cytokeratin 5/6 (CK5/6; 1:200 dilutions, anti-mouse), anti-p40 (1:200 dilutions, anti-rabbit), anti-phosphorylated Akt (pAkt; 1:2000 dilutions, anti-rabbit), anti-Akt (1:1000 dilutions, anti-rabbit), anti-phosphorylated Erk1/2 (pErk; 1:1000 dilutions, anti-rabbit), anti-Erk 1/2 (Erk; 1:1000 dilutions, anti-rabbit). Secondary antibodies: Histology and imaging_Enzyme-labeled goat Anti-Rouse / Rabbit IgG Polymer (working fluid). All antibodies used in the study are validated for species by manufacturer.

Animals and other organisms

Policy information about studies involving animals; ARRIVE guidelines recommended for reporting animal research

Laboratory animals	NOD-Prkdscidl12rgem2Idmo (NPI) mice from Beijing IDMO Co., Ltd. Six- to eight-week-old, male
Wild animals	No wild animals were used.
Field-collected samples	No field collected samples were used.
Ethics oversight	All animal studies were performed with approval from the Animal Care and Use Committee of the People's Hospital of Peking University.

Note that full information on the approval of the study protocol must also be provided in the manuscript.

Human research participants

Policy information about studies involving human research participants

Population characteristics	The detailed information about all the participants can be found in Supplementary table 1 and 2.
Recruitment	Main inclusion criteria include patients with clinically local advanced or metastatic lung cancer, aged 18 years or older, fresh tissues available through either biopsy or surgical resection of the primary or metastatic lesions. There was no bias on patient selection and the inclusion criteria did not impact the results.
Ethics oversight	The study was approved by the ethical review boards of the Peking University People's Hospital

Note that full information on the approval of the study protocol must also be provided in the manuscript.

Flow Cytometry

Plots

Confirm that:

- [x] The axis labels state the marker and fluorochrome used (e.g. CD4-FITC).
- [x] The axis scales are clearly visible. Include numbers along axes only for bottom left plot of group (a 'group' is an analysis of identical markers).
- [x] All plots are contour plots with outliers or pseudocolor plots.
- [x] A numerical value for number of cells or percentage (with statistics) is provided.

Methodology

Sample preparation	LCOs were treated with 10 μM of gemcitabine and vehicle (0.1% DMSO) for 24 h, followed by incubation with 1x EdU medium for 2 h. After that, LCOs were harvested and dissociated into single cells using an Organoid Harvesting Solution (R&D Systems) combined with TrypLE (Life Technologies). Dissociated single cells were fixed in 4% paraformaldehyde for 15 min and permeated with 0.3% Triton-X100 for 12 min at room temperature. After a wash with 3% BSA, cells were stained using the BeyoClick™ EdU Cell Proliferation Kit (Beyotime Biotechnology).
Instrument	BD FACS Aria II flow cytometry
Software	Flowjo software
Cell population abundance	The fraction of cells in the S phase was analyzed (13.9% in the non-treated sample and 0 in the Gefitinib treated sample).
Gating strategy	The cell cycle was analyzed by first circling the cell group with forward (FSC) and lateral (SSC), then removing the adherent cells with the W (signal width) / A (signal area) , finally, the cell cycle was analyzed by histogram of the fluorescence channel.

- [x] Tick this box to confirm that a figure exemplifying the gating strategy is provided in the Supplementary Information.
